# Clinical features, treatment and prognosis of MuSK antibody-associated myasthenia gravis in Northwest China: a single-centre retrospective cohort study

**DOI:** 10.1186/s12883-021-02439-7

**Published:** 2021-11-04

**Authors:** Sijia Zhao, Kai Zhang, Kaixi Ren, Jiarui Lu, Chao Ma, Cong Zhao, Zhuyi Li, Jun Guo

**Affiliations:** 1 Department of Neurology, Tangdu Hospital, Air Force Medical University, Xi’an, 710038 Shaanxi Province China; 2 Department of Intensive Care Unit, Xi’an No.3 Hospital, Xi’an, 710018 Shaanxi Province China; 3 Department of Cardiology, Tangdu Hospital, Air Force Medical University, Xi’an, 710038 Shaanxi Province China; 4 Department of Neurology, Air Force Medical Center of PLA, Beijing, 100142 China

**Keywords:** Muscle-specific kinase, Myasthenia gravis, Rituximab, Tacrolimus, Azathioprine

## Abstract

**Background and purpose:**

To summarize the clinical characteristics of patients with muscle-specific kinase antibody-associated myasthenia gravis (MuSK-MG) and to evaluate the therapeutic responses to different treatment regimes.

**Methods:**

Eighteen MuSK-MG patients admitted in our department between October 2017 and September 2020 were included. Clinical parameters were collected and the responses to different immunosuppressive drugs were assessed by MGFA Postintervention Status (MGFA-PIS). Meanwhile, the correlation between QMG scores and MuSK antibody titers were analyzed and MuSK antibody (MuSK-ab) titers were compared before and after therapy based on different immunosuppressive treatment regimes.

**Results:**

Female predominance (ratio of females to males, 15:3) was evident in the study population, with the average onset age of (40.28 ± 18.57) years and the median disease course of 30.50 months (interquartile range [IQR], 17.50–44.75 months). Ocular manifestation was the most common onset symptom (11/18; 61.11%), and mild symmetrical ptosis was most frequent. Bulbar symptoms had the highest incidence of 88.89% over the entire disease course. Abnormal responses to RNS test were recorded most frequently on the musculus deltoideus (83.33%). All patients were treated with prednisone (Pred) alone or plus azathioprine (AZA), tacrolimus (TAC) or low-dose rituximab (RTX), and 17 (94.44%) of them achieved a favorable outcome defined as minimal manifestation (MM) or better. In general, an obvious positive correlation between QMG score and MuSK-ab titer (*r* = 0.710, *P* < 0.001) were found in all patients. A more significant reduction of MuSK-ab titers was observed in patients receiving TAC or RTX plus Pred than those receiving AZA plus Pred.

**Conclusions:**

The prominent clinical manifestations of ocular and bulbar muscles involvements, together with abnormal RNS response mostly recorded on the musculus deltoideus and better efficacy associated with TAC or low-dose RTX plus Pred, provide a more exhaustive picture of MuSK-MG, particularly in Northwest China.

## Introduction

Since the first description of muscle-specific kinase antibody (MuSK-ab) as a novel autoantibody in 2001 [1], MuSK antibody-associated myasthenia gravis (MuSK-MG) has been regarded as an MG subtype with unique molecular immunology underpinning the pathology and clinical characteristics. It accounts for about 4–5% of all MG patients and one-third of acetylcholine receptor antibody (AChR-ab)-negative MG patients [2, 3]. Unlike AChR antibody-associated MG (AChR-MG), MuSK-MG displays different clinical profile including prominent involvement of bulbar and cranial muscles and rapid progression with myasthenic crisis at an earlier stage of disease course and shows a limited response to conventional treatments such as acetylcholinesterase inhibitors (ACEI), intravenous immunoglobulin (IVIg) and thymectomy [4, 5].

Given the fact that the non-complement activating IgG4 subclass is dominant in MuSK-MG, a great number of studies have concentrated on the development of new treatment regimens over the past two decades. Among those, B cell depletion therapy with monoclonal antibodies, such as rituximab (RTX) has been demonstrated to provide robust and long-lasting efficacy [4, 6, 7]. However, several issues remain undetermined including the optimal doses for MuSK-MG and the timing of initial infusion. Moreover, the therapeutic efficacy and long-term outcome of other immunosuppressive agents such as tacrolimus (TAC) in the treatment of MuSK-MG has been rarely reported [8].

In this retrospective cohort study, we reviewed the medical records of all MuSK-MG patients admitted in our department from October 2017 to September 2020 and summarized the clinical characteristics of MuSK-MG. Responses to different treatment regimens were assessed according to MGFA Postintervention Status (MGFA-PIS). Meanwhile, dynamics of serum MuSK-ab titers were monitored for each treatment regimen. This study aims to characterize the overall clinical picture of this MG subtype, especially in Northwest China.

## Methods

### Study protocol and data collection

We conducted a retrospective cohort study enrolling all MuSK-MG patients admitted in the Department of Neurology, Tangdu Hospital of Air Force Medical University between October 2017 and September 2020. Data including onset age, gender, clinical manifestations, MuSK antibody (MuSK-ab) titers, response to repetitive nerve stimulation (RNS) test, and medication history were collected. Serum levels of MuSK-ab were detected by a commercially available radioimmunoprecipitation assay kit (RSR Ltd., Cardiff, United Kingdom). According to the manufacturer’s instructions, ^125^I-labelled MuSK was used and the cutoff value was preset at 0.05 nmol/L. Values of > 0.05 nmol/L were defined as MuSK-ab positivity as recommended by a previous study [9]. RNS test was performed in four muscles: abductor digiti minimi after ulnar nerve stimulation, orbicular oculi after facial nerve stimulation, trapezius after accessory nerve stimulation, and musculus deltoideus after axillary nerve stimulation. Amplitude decrement of > 10% in compound muscle action potential (CMAP) was considered as a positive response. The classification of Myasthenia Gravis Foundation of America (MGFA) and Quantitative Myasthenia Gravis (QMG) scores were used to determine the severity of disease and the efficacy of different immunosuppressive treatments. Clinical outcomes were assessed by MGFA Postintervention Status (MGFA-PIS) and a favorable outcome was defined as the achievement of minimal manifestations (MM) or better, including complete stable remission (CSR), pharmacologic remission (PR) and MM.

### Immunosuppressive treatment regimes

The regimen of low-dose RTX was 100 mg per week for 3 consecutive weeks as induction treatment. Depletion of B cells was defined as the percentage of CD19^+^ B cells lower than 1% of total lymphocyte count and CD19^+^CD27^+^ memory B cells lower than 0.05%. The percentage of B cells was monitored every 6 months, and reinfusion (RTX, 100 mg) was given as maintenance treatment when B cells repopulation (defined as percentages of CD19^+^ B cells > 1% and/or CD19^+^CD27^+^ memory B cells > 0.05%) occurred. AZA was administrated at 2–3 mg/kg/day. TAC was administrated at an oral dose of 3 mg/day. Intravenous methylprednisolone therapy (IVMP) was administrated at 1 g/day for 3 days followed by 500 mg/day for 2 days for severe generalized condition according to the suggestions from the experienced neurologists. Oral prednisone (Pred) was given at an initial dose of 0.6–0.8 mg/kg per day with slow tapering of 5 mg every month.

### Statistical analysis

Data was presented as number with percentage, mean with standard deviation (SD) or median with interquartile range (IQR) where appropriate. The correlation between QMG score and MuSK-ab titer was analyzed using the Spearman’s rank correlation test and a value of *P* < 0.05 was considered statistically significant.

## Results

### Clinical characteristics

A total of 274 MG patients were reviewed and 18 MuSK-MG patients were confirmed with a prevalence of 6.57% in total MG. We then outlined the detailed demographic information and clinical characteristics of the 18 MuSK-MG patients (Table 1) and then conducted a pooled analysis as shown in Table 2. The enrolled MuSK-MG patients had a female predominance (3 males, 15 females) with an average onset age of (40.28 ± 18.57) years. The median disease course was 30.50 months (interquartile range [IQR], 17.50–44.75 months). At diagnosis, serum MuSK-ab was detected with a mean level of (1.50 ± 2.80) nmol/L. 11 (61.11%) of these patients showed a positive response to pyridostigmine test. At the disease onset, ocular manifestation was the most common complaint and was seen in 11 (61.11%) patients. Of them, 8 presented with mild ptosis, 1 with diplopia, and 2 with combined symptoms. Of note, ptosis was generally symmetrical (8 out of 10 cases). However, over the entire disease course, involvement of bulbar muscles was the most common manifestation as revealed in 16 (88.89%) patients. All patients underwent RNS test and 15 (83.33%; 2 males and 13 females) of them had abnormal responses on at least one nerve. The highest proportions of abnormal RNS responses were recorded in the musculus deltoideus (83.33%) followed by the orbicular oculi (66.67%), the trapezius (44.44%), and the abductor digiti minimi (16.67%). No muscle atrophy was observed. Three (16.67%) patients had thymic abnormalities on chest CT scan, and 3 of them had other autoimmune diseases.

**Table 1:** Detailed demographic and clinical information of the enrolled 18 MuSK-MG patients

Patient No.	Gender	Onset age (y)	Disease duration (m)	Onset symptoms	Pyridostigmine test	MuSK-ab at diagnosis (nmol/L)	RNS test				Thymic abnormalities on chest CT	Comobidities
							Musculus deltoideus	Trapezius	Orbicular oculi	Abductor digiti minimi		
1	F	25	58	3	+	1.21	+	–	+	–	–	–
2	F	25	47	3	+	1.10	+	+	+	+	–	Hypothyroidism, thyroid cancer
3	F	69	70	1,2,3	–	0.66	+	–	+	–	–	Hypertension, DM
4	F	46	42	1	+	1.20	+	–	+	–	–	–
5	F	49	39	1,2	+	1.17	–	–	–	–	+	Hypothyroidism
6	M	17	25	1	+	1.08	+	–	+	–	–	–
7	F	16	18	2	–	0.51	+	+	+	–	–	–
8	F	45	9	1,2	–	0.66	+	–	+	–	–	–
9	F	37	196	1,2	+	1.73	+	+	–	–	–	–
10	F	49	10	1,2,3	–	0.63	+	–	+	–	–	Hypertension
11	F	27	39	3	+	1.10	+	+	+	–	–	–
12	F	36	44	1,2	+	0.57	+	+	+	+	–	Hypothyroidism
13	F	28	27	2,3	–	0.77	+	–	–	–	–	–
14	M	28	16	2	–	0.48	+	+	–	–	+	–
15	F	44	18	3	–	0.51	+	+	+	–	–	–
16	F	31	24	1	+	0.34	+	+	+	+	–	–
17	M	76	34	1,2	+	12.64	–	–	–	–	–	–
18	F	77	1	1	+	0.60	–	–	–	–	+	Coronary heart disease, lung nodules, DM

*Abbreviations*: *MuSK-ab* Muscle-specific kinase antibody, *RNS* Repetitive nerve stimulation, *CT* Computed tomography, *DM* Diabetes mellitus, *1* Ptosis and/or diplopia, *2* Bulbar symptoms, *3* Limbs symptoms, *No.* Number, *F* Female, *M* Male, *y* Year, *m* Month; +, positive; −, negative

**Table 2:** Pooled analysis of clinical characteristics of the 18 MuSK-MG patients

Variables	
Gender ratio, F:M	15:3
Onset age (y), mean ± SD	40.28 ± 18.57
Disease duration (m), median (IQR)	30.50 (17.50–44.75)
Onset symptoms	
Ocular, *n* (%)	11 (61.11)
Bulbar, *n* (%)	10 (55.56)
Limbs, *n* (%)	7 (38.89)
Serum MuSK-ab titer (nmol/L) at diagnosis, mean ± SD	1.50 ± 2.80
RNS test positive	
Musculus deltoideus, *n* (%)	15 (83.33)
Trapezius, *n* (%)	8 (44.44)
Orbicular oculi, *n* (%)	12 (66.67)
Abductor digiti minimi, *n* (%)	3 (16.67)
Any muscles	15 (83.33)
Pyridostigmine test positive, *n* (%)	11 (61.11)
Thymic abnormalities on chest CT, *n* (%)	3 (16.67)
Symptoms involved over the disease course	
Ocular, *n* (%)	13 (72.22)
Bulbar, *n* (%)	16 (88.89)
Limbs, *n* (%)	7 (38.89)
Myasthenic crisis, *n* (%)	5 (27.78)
QMGs before therapy, mean ± SD	12.83 ± 5.61
QMGs at last follow-up, mean ± SD	0.17 ± 0.51

*Abbreviations*: *QMGs* Quantitative Myasthenia Gravis score, *MuSK-ab* Muscle-specific kinase antibody, *RNS* Repetitive nerve stimulation, *CT* Computed tomography, *F* Female, *M* Male, *Y* Year, *m* Month, *n* Number of patients, *%* Percentage, *SD* Standard deviation, *IQR* Interquartile range

### Treatment and prognosis

**Fig. 1:**
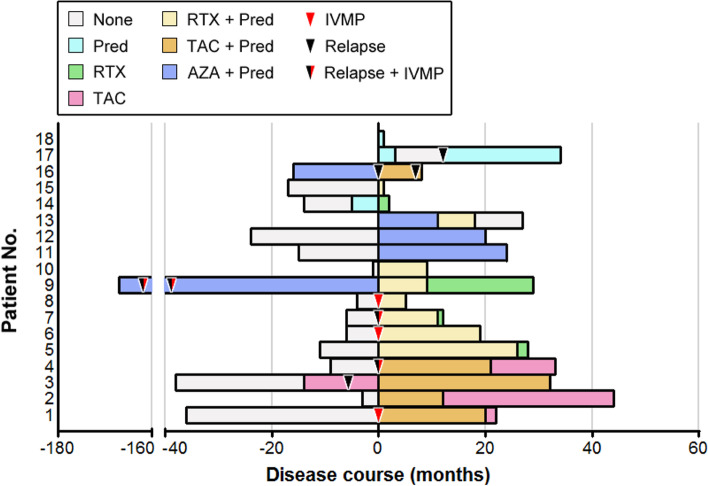
Detailed treatment regimes of the enrolled 18 MuSK-MG patients during the disease course. On the *x*-axis, 0 indicates the time point when MuSK-MG diagnosis was confirmed at our department. *Abbreviations:* Pred, Prednisone; AZA, azathioprine; TAC, tacrolimus; RTX: rituximab; IVMP: intravenous methylprednisolone therapy

Figure 1 showed all immunosuppressive treatment regimens for the enrolled MuSK-MG patients over the entire course of disease, and the detailed information associated with disease severity and treatment responses were revealed in Table 3. All patients were mainly treated with the following therapeutic protocols: prednisone monotherapy (Pred), Pred plus azathioprine (AZA), Pred plus tacrolimus (TAC), and Pred plus rituximab (RTX). Overall, 17 (94.44%) of the patients eventually achieved a favorable outcome. During the immunotherapy period, a total of 9 relapses occurred in 6 patients, with 3 relapses in one patient when receiving AZA plus Pred and 2 relapses in another one patient when receiving AZA plus Pred and TAC plus Pred therapies, respectively. No serious adverse events associated with immunosuppressive agents were observed in all the patients.

**Table 3:** Detailed information associated with disease severity and treatment responses of the 18 enrolled MuSK-MG patients

Patient No.	Immunotherapy history	QMGs before therapy	MGFA before therapy	Maximum QMGs	Maximum severity MGFA	QMGs at last follow-up	MGFA-PIS at last follow-up	Immunotherapy at last follow-up	MuSK-ab at last follow-up (nmol/L)	No. of myasthenic crisis	Time from onset to crisis (m)
1	TAC, Pred	22	IIIb	28	V	0	PR	TAC (3 mg/d)	0.001	1	34
2	TAC, Pred	15	IIIb	15	IIIb	0	PR	TAC (3 mg/d)	0.001	0	NA
3	TAC, Pred	16	IIIb	16	IIIb	0	MM	TAC (3 mg/d) Pred (20 mg/d)	0.20	0	NA
4	TAC, Pred	10	IIb	13	IIb	0	PR	TAC (3 mg/d)	0.46	0	NA
5	RTX, Pred	12	IIb	12	IIb	0	PR	RTX (100 mg/6 m)	0.22	0	NA
6	RTX, Pred	6	I	6	I	0	MM	RTX (100 mg/5 m) Pred (5 mg/d)	0.26	0	NA
7	RTX, Pred	11	IIb	11	IIb	0	PR	RTX (100 mg/6 m)	0.001	0	NA
8	RTX, Pred	20	V	20	V	0	MM	RTX (100 mg/6 m) Pred (5 mg/d)	0.20	1	3
9	AZA, Pred, RTX	7	V	26	V	0	PR	RTX (100 mg/6 m)	0.47	3	16^a^
10	RTX, Pred	19	V	19	V	0	MM	RTX (100 mg/6 m) Pred (15 mg/d)	0.25	1	2
11	AZA, Pred	15	IIb	22	V	0	PR	Pred (5 mg/d)	0.45	1	15
12	AZA, Pred	6	IIb	6	IIb	2	I	Pred (10 mg/d)	0.51	0	NA
13	AZA, Pred, RTX	8	IIb	14	IIIb	0	CSR	None	0.37	0	NA
14	Pred, RTX	12	IIb	12	IIb	0	PR	RTX (100 mg/6 m)	ND	0	NA
15	RTX, Pred	20	IIIb	20	IIIb	0	PR	RTX (100 mg/6 m) Pred (20 mg/d)	ND	0	NA
16	AZA, Pred, TAC	18	IIb	18	IIb	1	MM	TAC (3 mg/d) Pred (25 mg/d)	ND	0	NA
17	Pred	11	IIb	16	IIIb	0	PR	Pred (10 mg/d)	ND	0	NA
18	Pred	3	I	3	I	0	MM	Pred (25 mg/d)	ND	0	NA

*Abbreviations*: *QMGs* Quantitative Myasthenia Gravis score, *MGFA* Myasthenia Gravis Foundation of America, *MGFA-PIS* MGFA Postintervention Status, *MuSK-ab* Muscle-specific kinase antibody, *Pred* Prednisone, *AZA* Azathioprine, *TAC* Tacrolimus, *RTX* Low-dose rituximab, *CSR* Complete stable remission, *PR* Pharmacologic remission, *MM* Minimal manifestations, *I* Improved, *No.* Number, *NA* Not applicable, *ND* Not done, *m* Month. ^a^ represents the interval from disease onset to the first crisis

Specifically, 3 patients were initially treated with prednisone alone and two of them achieved complete clinical remission with the QMG score of 0 at the last follow-up. The residual one asked for a switch to low-dose RTX monotherapy since MuSK-MG was diagnosed and also achieved complete clinical remission. Initial AZA plus Pred therapy was given to 5 patients, but only one achieved a favorable outcome at the last follow-up with AZA plus low-dose prednisone (10 mg/day). Other 3 experienced frequent relapses and/or requirement of a high maintenance dose of Pred, and two of them achieved a favorable outcome after switching to RTX and Pred was discontinued eventually. One was switched to TAC and clinical symptoms were well alleviated, however, Pred at 25 mg/day was required and tapering was associated with an exacerbation of symptoms. She was advised to switch to receive RTX at the last follow-up. Besides, the residual one achieved an unfavorable outcome with obvious bulbar symptoms left, but she refused to receive other immunosuppressive agents because of the concerns about the high costs and potential drug-associated adverse events. Moreover, initial low-dose RTX or TAC plus Pred therapy also resulted in a favorable outcome, and no switch treatment occurred. Until the last follow-up, complete withdrawal of Pred had been obtained in 2 patients initially with low-dose RTX plus Pred and 3 with TAC plus Pred.

### MuSK antibody titer monitoring

**Fig. 2:**
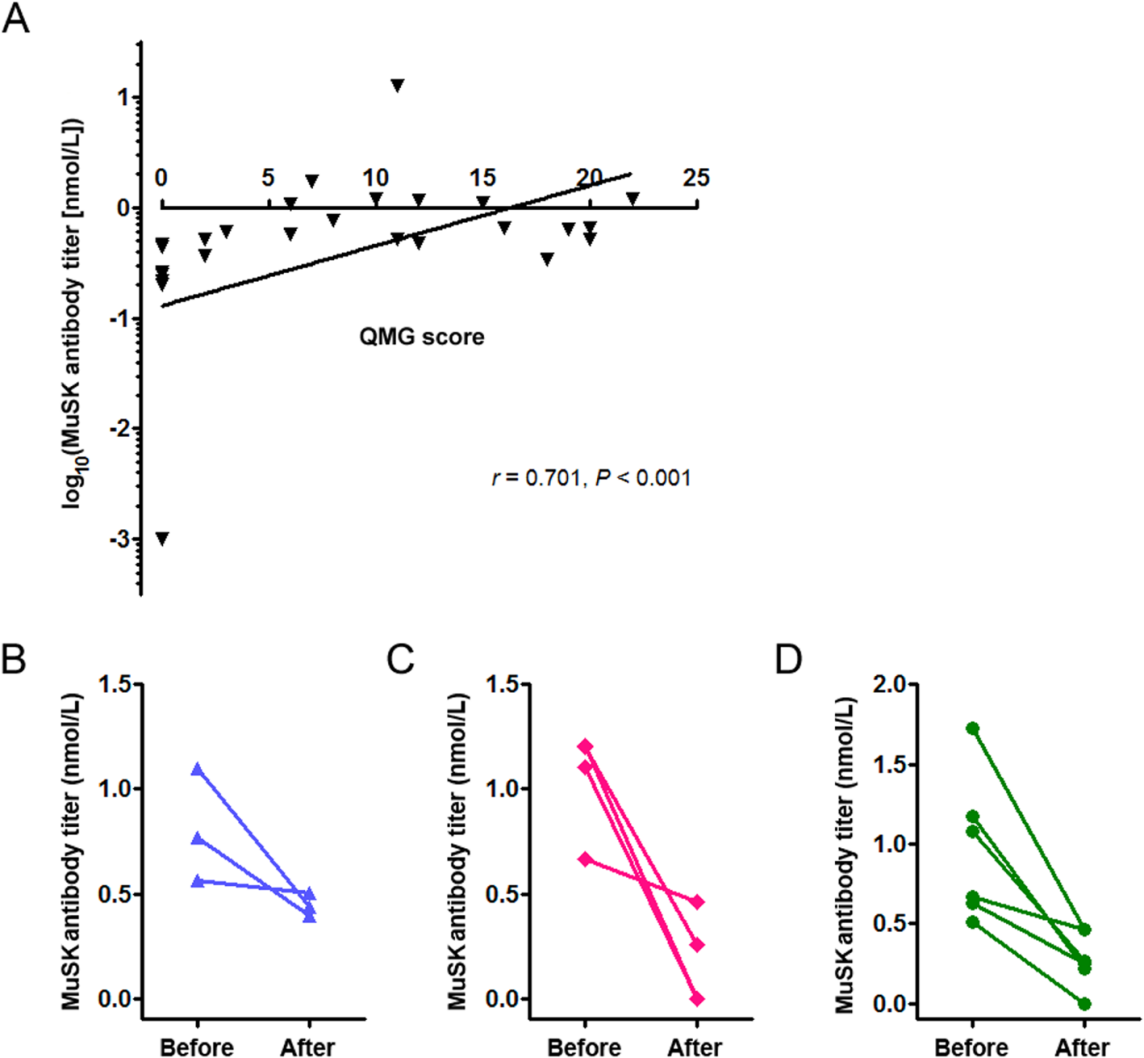
Correlation analysis of MuSK-ab titer with QMG score and dynamic monitoring of antibody titers with different treatment regimes. **A** An obvious positive correlation between MuSK-ab titer and QMG score was demonstrated. Data from 18 pre-treatment patients and 13 post-treatment patients (*n* = 31) were included into the statistical analysis. **B** The mean Musk-ab titer was reduced by 45.68% in 3 patients receiving AZA plus Pred compared with that before treatment. **C** The mean MuSK-ab titer was reduced by 82.69% in 4 patients receiving TAC plus Pred compared with that before treatment. **D** The mean MuSK-ab titer was reduced by 76.04% in 6 patients receiving low-dose RTX plus Pred compared with that before treatment. *Abbreviations:* MuSK-ab: muscle-specific kinase antibody; Pred, Prednisone; AZA, azathioprine; TAC, tacrolimus; RTX: rituximab

Given the potential correlation of MuSK-ab titer with the severity of disease, we investigated the data on QMG scores and MuSK-ab titers from all pre-treatment patients and 13 post-treatment patients, and found an obvious positive correlation between the two parameters (*r* = 0.710, *P* < 0.001; Fig. 2A). Then, we compared the changes of MuSK-ab titers before and after treatment in 13 patients who have relevant data of both time points. As shown in Fig. 2B–D, compared with the pre-treatment mean antibody titers, the most significant decrease was observed in patients receiving TAC plus Pred (82.69% reduction), followed by low-dose RTX plus Pred (76.04% reduction) and AZA plus Pred (45.68% reduction).

## Discussion

It is well demonstrated that activation of MuSK triggers AChR clustering on the postsynaptic membrane of the neuromuscular junction [10]. MuSK-ab primarily is of non-complement activating IgG4 subclass [11] and acts by interrupting the interaction of MuSK, LRP4 and collagen Q [12–14]. The pathological mechanism distinct from that of AChR-ab might partially underpin the unique profile of MuSK-MG. In this study, we observed a significant female predominance and bulbar symptoms had the highest incidence over the entire disease course, which was consistent with prior studies [3, 4, 6]. Meanwhile, of special note were ocular symptoms especially symmetrical ptosis as previously reported [15], occupying the first position at the disease onset of our patient cohort, although the symptoms were often subtle and could be easily neglected in the earlier stage of the disease. Therefore, careful attention to the characteristic manifestations should be paid by clinicians to facilitate a prompt diagnosis. A recent study has proposed that ocular myasthenia may be considered as a new clinical phenotype for MuSK-MG [16]. This rare phenotype was also found in our cohort (case 6) and another cohort from Northeast China [17], suggesting the requirement to notice this specific phenotype in the Han Chinese population. In this study, case 6 had pure ocular symptoms for more than 2 years and was determined as ocular MG, whereas the possibility of secondary progression cannot be excluded since a slow disease progression has been confirmed in such patients from a recent MuSK-MG study by Zhang et al. [17]. In addition, in contrast to previous studies [17–19], muscle atrophy is not distinct in our patient cohort, which might be partly attributed to a short interval from onset to MG diagnosis and early initiation of interventions. A longer follow-up is needed to clarify this discrepancy.

Prior evidence has revealed that the patterns of RNS responses play an important role in diagnosing MuSK-MG and distinguishing it from other MG subtypes, in which abnormal RNS responses in facial muscles but normal in limbs are independently associated with MuSK-MG [20]. In another study by Oh et al. [21], the highest proportion of abnormal RNS responses was shown in facial muscles, also reflecting the propensity for facial muscle involvement in this unique subtype. Similarly, a high frequency of RNS abnormalities in the orbicular oculi is also observed in this study, whereas musculus deltoideus that was not tested in prior studies was the most susceptible in our patient cohort. It should be verified whether the inclusion of musculus deltoideus in RNS protocols may increase the diagnostic yield of MuSK-MG. Conversely, negative results on the RNS test can make it more difficult to diagnose MuSK-MG since this subtype also exhibits unique clinical profiles including a lack of diurnal symptom fluctuations and a limited response or unresponsiveness to ACEI. In this situation, the detection of MuSK-ab would be crucial for a definite diagnosis. Besides, thymic hyperplasia or thymoma is rarely reported in MuSK-MG, and thymectomy shows limited responses due to the minimal thymic histological alterations documented in such patients [22]. Likewise, thymic abnormalities were found in only 3 patients when the chest CT scan was performed, exhibiting an increased anterior mediastinal fat density accompanied by multiple internal linear soft tissues. Unfortunately, all three patients did not receive thymectomy. Thus, the histological changes remained undetermined and would be monitored during follow-up.

The treatments of MuSK-MG are usually compared with those of AChR-MG since distinct pathogenic mechanisms underlie the two subtypes [2, 3, 23]. It has been generally acknowledged that MuSK-MG shows limited responses to conventional immunosuppressive treatments including IVIg, AZA, and thymectomy, whereas more robust responses are obtained with prednisone, RTX, and plasma exchange [2]. Similar to previous studies [6, 7], in our cohort over half of the patients who were initially treated with AZA plus Pred failed to achieve a satisfactory outcome. Conversely, two patients switching from AZA to low-dose RTX plus Pred achieved a favorable outcome eventually. Of note, the dosages of RTX in the treatment of MuSK-MG are diverse and there is a lack of definite consensus on optimal RTX dosage [23–25]. Although a higher dose of RTX (e.g., 375 mg/m^2^ for each infusion) has been often proposed in previous studies [26, 27], a modified dose of RTX used in this study, irrespective of initial or switch treatments, has proven to be effective and safe for this subtype based on the achievement of QMG score of 0 in all the 9 patients receiving this treatment regime and no occurrence of serious adverse events. Meanwhile, 77.8% of patients (7/9) received a dose of oral Pred of 5 mg/day or complete withdrawal at the last follow-up. These findings have implications for the application of low-dose RTX as a cost-effective treatment protocol for MuSK-MG.

To date, few studies explore the efficacy and safety of TAC in MuSK-MG [8, 28], although it has been regarded as an effective treatment for AChR-MG [22, 29]. In our cohort, 5 patients received TAC plus Pred therapy. Of them, 3 with initial TAC plus Pred achieved a QMG score of 0 at the last follow-up, and a similar result was obtained in another Chinese MuSK-MG cohort with short-term follow-up [17]. Another patient with initial TAC monotherapy underwent the fluctuation of symptoms, whereas complete remission was obtained when in combination with Pred and a stable clinical status was maintained during the subsequent tapering of Pred, suggesting the importance of TAC plus Pred therapy initiated at the earlier stage of the disease. Besides, the residual one patient switching from AZA experienced one more relapse, possibly implying a poorer efficacy of delayed TAC therapy compared with early treatment. The hypothesis requires confirmation by prospective studies with a large number of patients. Collectively, TAC may provide a promising choice of treatment for MuSK-MG.

Recent evidence has shown that serum MuSK-ab titer is associated with the severity of disease [30, 31], and a reduction of antibody titer might be a potential predictor for a favorable clinical response to RTX [32]. Similar to prior studies [18, 26, 27], a reduction of antibody titer was obtained to varying degrees in our cohort with different treatment protocols, which was accompanied by the improvement of clinical status demonstrated with QMG scores. Patients receiving TAC plus Pred achieved the most remarkable decrease of MuSK-ab titer, followed by those receiving low-dose RTX plus Pred and AZA plus Pred. These findings were well consistent with the remission of clinical status and indicate that dynamic monitoring of MuSK-ab titer might predict the responses to immunosuppressive treatments and guide adjustment of treatment regimes. However, conclusions drawn from this small sample size and heterogeneously treated patient sample limited the significance, and future studies are needed to confirm the hypothesis.

In conclusion, MuSK-MG exhibits unique clinical profiles including female predominance and the most frequently abnormal response of musculus deltoideus on the RNS test. In addition to the well-recognized bulbar involvement, mild ocular symptoms serve as another common manifestation at the onset. Low-dose RTX or TAC plus Pred could be considered as a promising first choice of treatment given its favorable efficacy and safety profile. In the future, high-quality trials with larger samples and longer follow-up are required to clarify the long-term efficacy and safety of different treatment regimes.

## Data Availability

The data sets in this study are available from the corresponding author on reasonable request.
